# 

**Published:** 1872-04

**Authors:** 


### CONTENTS.

### COMMUNICATIONS.

Severe Ptyalism from a Dental Operation Without Amalgam ........141
Soft Foil vs. Adhesive Foil ........142
Poisoned by Mercury from a Filling ........145
A Word for Nitrous Oxide ........146
Correspondence ........148
Address by Dr. James Taylor ........150
Discussion in the Mississippi Valley Dental Society ........159
Green's Improved Burr Engine ........183

### PROCEEDINGS OF SOCIETIES.

Dental Society of New York ........180
Meeting of the Eastern Dental Association ........184
The Annual Meeting of the Lake Erie Dental Association ........184

### OBITUARY.

Dr. Elisha Baker ........181

### EDITORIAL.

Departures—Professional ........185
Celluloid Base ........186
Spring Course ........187
The State Board of Examiners ........187

